# Supplementary figures and images for: Evaluation of the preclinical analgesic efficacy of naturally derived, orally administered oil forms of Δ9-tetrahydrocannabinol (THC), cannabidiol (CBD), and their 1:1 combination
Source: PLoS One. 2020 Jun 4;15(6):e0234176. doi: 10.1371/journal.pone.0234176 (PMC7272035; doi:10.1371/journal.pone.0234176)

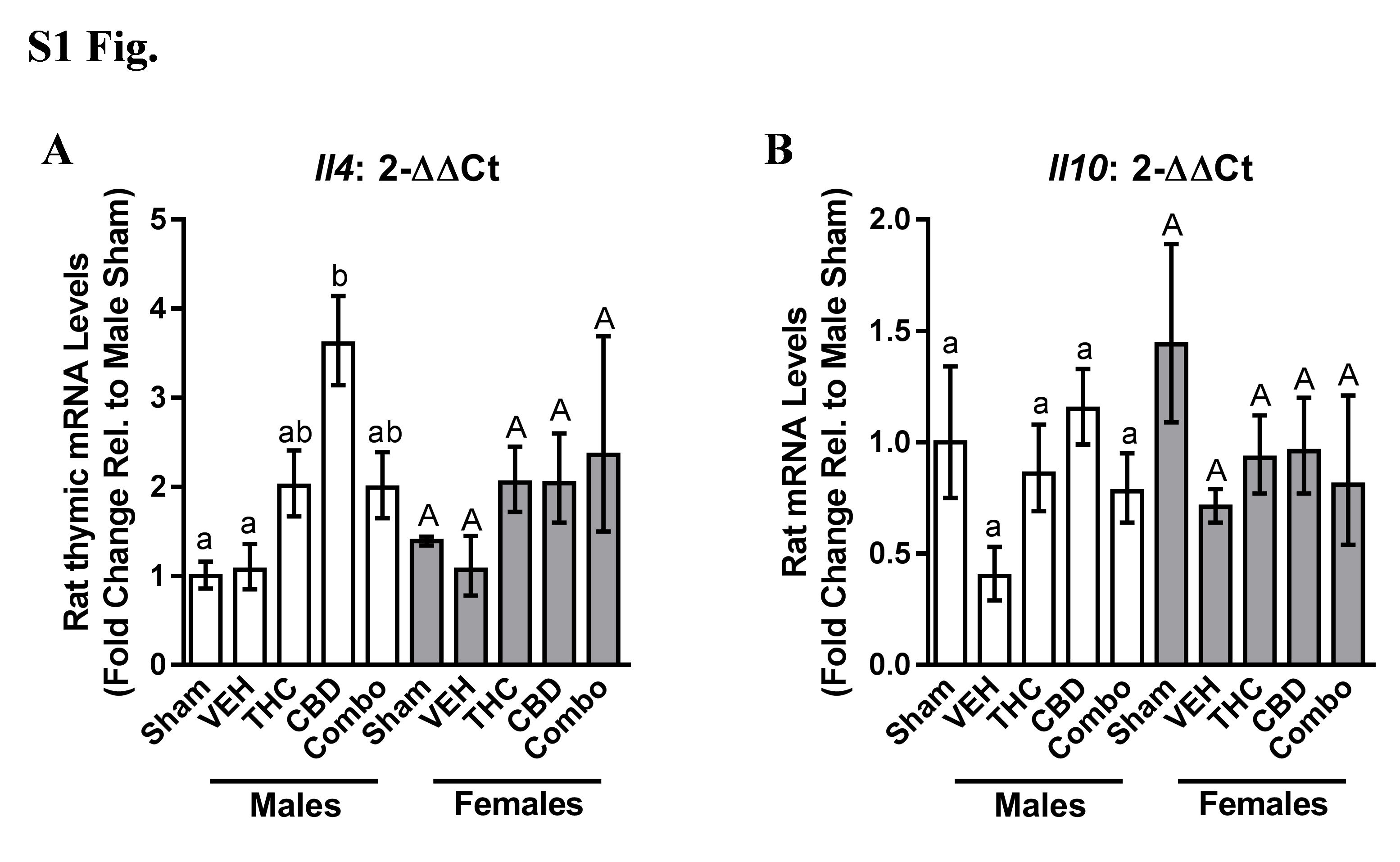

Supplement: S1 Fig — Thymic A) Il4 and B) Il10 mRNA levels were examined at experimental endpoint by RT-qPCR in males and females implanted with a sciatic nerve cuff that had undergone early cannabinoid treatment. Data are expressed as the mean ± SEM for each treatment group, and is presented relative to male sham-treated rats, with statistical significance analyzed by 1-way ANOVA with a Tukey’s post-hoc comparison. Different letters (male comparison: a, b, etc.; female comparison: A, B, etc.) represent differences between groups (P<0.05). VEH: vehicle (MCT oil), Combo: 1:1 combination of THC and CBD. (TIF) [file pone.0234176.s001.tif]
