# Supplementary material for: Evaluation of the preclinical analgesic efficacy of naturally derived, orally administered oil forms of Δ9-tetrahydrocannabinol (THC), cannabidiol (CBD), and their 1:1 combination
Source: PLoS One. 2020 Jun 4;15(6):e0234176. doi: 10.1371/journal.pone.0234176 (PMC7272035; doi:10.1371/journal.pone.0234176)
Supplement: Attachment — Submitted filename: Response to Reviewers.docx [file pone.0234176.s002.docx]

**Response to Editorial Comments/ Redress of Reviewers’ Comments**

PONE-D-20-02929: Evaluation of the preclinical analgesic efficacy of naturally derived, orally administered oil forms of Δ9-tetrahydrocannabinol (THC), cannabidiol (CBD), and their 1:1 combination

**Journal Requirements:**

When submitting your revision, we need you to address these additional requirements. Please ensure that your manuscript meets PLOS ONE's style requirements, including those for file naming. The PLOS ONE style templates can be found at

https://journals.plos.org/plosone/s/file?id=wjVg/PLOSOne_formatting_sample_main_body.pdf and

https://journals.plos.org/plosone/s/file?id=ba62/PLOSOne_formatting_sample_title_authors_affiliations.pdf

Please note that the final manuscript file in which tracked changes have been accepted (‘Manuscript’) adheres to the requested style requirements outlined in the pdf files above.

While revising your submission, please upload your figure files to the Preflight Analysis and Conversion Engine (PACE) digital diagnostic tool, https://pacev2.apexcovantage.com/. PACE helps ensure that figures meet PLOS requirements. To use PACE, you must first register as a user. Registration is free. Then, login and navigate to the UPLOAD tab, where you will find detailed instructions on how to use the tool. If you encounter any issues or have any questions when using PACE, please email us at figures@plos.org. Please note that Supporting Information files do not need this step.

We have submitted all of the revised Figs 1-4 and the S1 Fig to PACE, and each one passed inspection (green check-mark next to all five uploaded files).

1. We note that you have included the phrase “data not shown” in your manuscript. Unfortunately, this does not meet our data sharing requirements. PLOS does not permit references to inaccessible data. We require that authors provide all relevant data within the paper, Supporting Information files, or in an acceptable, public repository. Please add a citation to support this phrase or upload the data that corresponds with these findings to a stable repository (such as Figshare or Dryad) and provide and URLs, DOIs, or accession numbers that may be used to access these data. Or, if the data are not a core part of the research being presented in your study, we ask that you remove the phrase that refers to these data.

We have removed this phrase from the revised manuscript, as it is not a core part of the research being presented in our study.

2. Please amend your list of authors on the manuscript to ensure that each author is linked to an affiliation. Authors’ affiliations should reflect the institution where the work was done (if authors moved subsequently, you can also list the new affiliation stating “current affiliation:….” as necessary).

We have amended the author list to ensure that each author is linked to the appropriate institution at the time of submission. Please note that we have also updated the order of the authors.

**Reviewers' comments:**

Please note that we have included line numbers in our redress of both reviewers’ comments to indicate where each change was made. These line numbers refer to the manuscript file with the tracked changes, so that the reviewers can see where in the original version the revisions were made.

**Reviewer #1:**

The authors state that "In preclinical models representing surgically induced peripheral nerve damage, nociceptors undergo sensitization, with afferent fibres developing patterns of ectopic neural discharge." This is not always true see ectopic discharges from injured nerve fibers are highly correlated with tactile allodynia only in early, but not late, stage in rats with spinal nerve ligation. Sun et al 2005.

We have changed this sentence to read: “In preclinical models representing surgically induced peripheral nerve damage, nociceptors undergo sensitization, and afferent fibres *may* develop patterns of ectopic neural discharge. As a consequence, a complex response in the spinal cord *may* be evoked that includes…” (lines 71-75). We would like to point out that, unlike the paper by Sun et al. 2005, which involves a spinal nerve ligation that involves the CNS, we are referring to models of peripheral nerve damage that are inflicted in the PNS, which may have different outcomes.

Authors state that "Initiating treatment at an early stage post-nerve injury (for example, PRIOR TO or directly after a surgery" but they provide no evidence for treatment helping before. The citation only includes early intervention. If NP is caused by aberrant neural discharge what can be done to prevent it from developing?

We have changed the sentence, removing the words “prior to”, to now read “Initiating treatment at an early stage post-nerve injury (for example, directly after a surgery)…” (lines 77-80).

In addition, we have added the following to address what could be done to prevent aberrant neuronal discharge post-nerve damage (lines 81-88): As proposed by Neil and Macrae (2009), reducing nociceptive input into the spinal cord during and after surgery may be “sensible” to attenuate sensitization of the nervous system, and anaesthetic techniques that accomplish this aim should be associated with reduced post-operative as well as chronic pain (4). In this context, research supports that local anaesthesia could provide potential benefits (5, 6). However, it may not be practically feasible to entirely eliminate persistent pain using this approach (7), and it has therefore been suggested that a variety of different analgesic agents be administered around the time of surgery.

Authors state "The predominant components of phytocannabinoid extracts, Δ9-tetrahydrocannabinol (THC) and cannabidiol (CBD), are able to modulate nociceptive thresholds and influence the release of pro-inflammatory molecules and chemokines by relevant non-neuronal cell types [reviewed in (18)]." The citation refers to an entire edition of a journal a more specific citation is needed for a direct claim.

We have added more specific citations; please refer to lines 115-116 of the revised Introduction: “…and THC suppresses the activities of T lymphocytes (22, 23), with CBD also altering the functional activity of these cells (24-26)”.

Authors do not mention CBDs direct agonism of 5HT1A which seems like a significant oversight.

We apologize for this oversight, and have added the following to the revised Introduction, lines 123-125: “In addition, it is important to note that other receptors, such as GPR55, NMDAR and GABAR, are also modulated by cannabinoids (30), with CBD directly agonising the 5-HT1A receptor (31).”

Authors do not justify the use of natural cannabis abstracts, which contain impurities. Why not use controlled synthetically produced compounds?

To address the use of natural cannabis extracts, we have now added the following to the revised Introduction (lines 134-141): “The rationale for orally administering naturally-extracted cannabis in MCT oil was that this particular formulation was used by patients at the Michael G. DeGroote Pain Clinic, McMaster University Medical Centre, as an alternative to smoking combusted cannabis or its vapor-based inhalation, not only to better control for dosing, but to avoid potential side-effects on lung health (37). While the entourage effect of other constituents present in the plant-based extracts, such as terpenes and flavonoids (38, 39), cannot be dismissed, each oral formulation used in the current study was highly enriched for either THC or CBD. (CannTrust).”

Authors have not provided any evidence or experiments to determine what THC and CBD are doing to produce the observed effects. Antagonist pharmacology to determine the exact nature of the effects of THC and CBD would significantly help the central argument of the paper.

We agree that the use of specific antagonists will be important to elucidate the effects of THC and CBD at specific receptors, and to better understand the molecular mechanisms underlying their actions in the context of NP. However, this was beyond the scope of the current investigation, which sought to determine the effects of these two cannabinoids in naturally derived oil extracts, administered orally, which are currently being used in the clinic by patients experiencing chronic pain.

**Reviewer #2: Overall Evaluation**

This is an interesting study to evaluate the effects of THC, CBD, and their combination in a rat model of peripheral neuropathic pain. The large sex differences are particularly interesting, but shortcomings in description of the Methods and Results, together with an unfocused discussion that does not adequately reference the existing literature on sex differences in the effects of cannabinoids in rodent models of neuropathic pain, must be addressed before this can be further considered for publication. Numerous additional minor comments are also interwoven with major comments throughout the following critique.

***1. The Methods are missing numerous key details.***

a. The cuff model is generally referred to as a preclinical model of peripheral neuropathic pain and not a preclinical model of “post-surgical neuropathic pain.” Otherwise, all of the preclinical models of PNP would have been referred to as models of “post-surgical neuropathic pain.”

We have addressed this issue by removing the term “post-surgical neuropathic pain” throughout the revised manuscript.

b. Detailed surgical methods are required. Was anesthesia used? Post-operative analgesics? Details of the cuff including material and size are needed. More fully describe the sham surgery, did it include isolation of the nerve?

This information has now been added to the Methods section; please refer to lines 169-181 of the revised manuscript.

c. Literature reference to the cuff model is needed in Methods

The citations that are relevant to the cuff model have now been added to the revised Methods; please see line 170.

d. Details of the feeding needles is needed in Methods. What was volume range of oral administration?

Details regarding the feeding needles have been added to the revised Methods, lines 20o-202. The volume that was orally administered was always 1 ml (line 202).

e. Please explain why only vF>9 was chosen as characteristic of baseline, (why not vF>8 for example?), and list numbers of animals removed from the study based on this criteria.

The value of 9 is used in our lab as a cut-off score. Any animal below a von Frey response of 9 g is considered to be a “basally poor responder” for this test, as it is important to have a consistent baseline prior to nerve cuff surgery in order to meaningfully evaluate changes that are indicative of post-nerve cuff-induced nociception (see more on this in our response to point f. below).

We generally order extra animals to ensure that our baseline criteria are met. We aimed to have at least n=10 animals that showed robust baseline behaviour in the von Frey test (n=5 per experiment, pooled over 2 independent experimental trials, given that it is difficult to handle too many rats in any given trial). Any rats that fall below this pre-experimental threshold are not included in the study (i.e. they are euthanized; never undergo surgery at the sciatic nerve cuff implantation site, and therefore, no further behavioural testing).

f. What is “responsive males” on page 8? Please indicate whether any animals were excluded for any reason after surgery.

“Responsive” refers to the animals that exhibited robust baseline behaviour prior to any surgical intervention or treatment, which should refer to both males and females, not only males as was stated in our initial submission. We have removed this term, as it does not add any significant meaning to the manuscript, nor affect any of the results. However, to clarify for the reviewer, responsive means that, prior to any surgery, an animal has a high baseline von Frey score (requiring a significant gram force, via the corresponding von Frey filament, to withdraw its paw). An animal with a low baseline score would indicate being sensitive even in the absence of a cuff that would later induce nociception, confounding the interpretation of the data, as in many cases, these “non-responsive” animals don’t end up exhibiting the classic drop in von Frey paw withdrawal force upon nerve impingement. Evaluating what happens to a cohort of “non-responsive” rats that are already hypersensitive upon arrival at the Central Animal Facility was not the aim of the current study.

We want to point out that NO animals were removed from the experiment after the nerve cuff surgery was carried out. In the very rare instance that an animal dies post-surgery, we remove the baseline data for that particular animal from the experiment.

g. Please indicate source vF filaments, indicate which ones were used, and briefly describe the up-down method used.

Please refer to the requested changes incorporated into the revised Methods that address this point, lines 219-231.

Clarify the statement “Data reflecting the paw withdrawal force”. Data is and should be plotted in gram force. “paw withdrawal force” is cumbersome.

We thank the reviewer for pointing out the use of this is cumbersome wording. The reviewer is correct that our data reflects the gram force. This has now been addressed, with the y-axis labeling in the corresponding figures (Fig. 1 and 2) adjusted accordingly.

h. At what time of day were behavioral experiments performed? By which experimenter? Did the “dedicated research staff” who handled the animals vary from day to day? Was the experimenter blind to treatment and if so what was the blinding procedure.

The time of day for the behavioural experiments is now clearly indicated in the revised Methods (lines 233-235). In addition, the following has been added (lines 205-210): “The research staff that carried out von Frey behavioural testing varied between experiment #1 and experiment #2, which were conducted approximately 6 months apart. However, within each given experiment, the same individuals handled all animals from one testing day to the next. Experimenters were blinded to treatment, with codes corresponding to the administered oil formulation ascribed to each cage, which were not shared with those conducting behavioural testing.”

i. Section on in vivo intracellular DRG recordings requires description of animal preparation including post-surgery date, anesthesia, surgery, and monitoring. Description of electrical stimulation are required throughout this section.

Please refer to lines 239-270 of the revised electrophysiological methods section, which addresses this point through addition of all of the requested details.

j. Criteria for fiber classification are required.

Please see lines 279-300 in the revised Methods section for the criteria used for the electrophysiological classification of DRG nerve fibers.

k. Description of stimuli including ‘muscle belly’ and ‘cutaneous’ is required.

Please see lines 294-300 in the revised Methods section regarding a more in-depth description of ‘muscle belly’ and ‘cutaneous’.

***2. The Results section***

a. It is controversial to use the term “chronic” for a behavior lasting a small number of weeks. Same for the term “allodynia” which may or may not be reflected by hypersensitivity to vF filaments. Use correct terminology, for example change “chronic mechanical allodynia” to “persistent mechanical hypersensitivity”

The term “chronic” has been, where appropriate, changed to either “persistent” or “sustained” throughout the revised manuscript (see tracked changes). The reviewer’s requested terminology for “persistent mechanical hypersensitivity” has now been applied throughout the text.

b. An appropriate rationale for choosing to focus on Week 3 and Week 6 would be helpful. Should definitely NOT have been chosen to subjectively determine any greater effect of one drug / drug combination over another. Alternative is to display area under the curve across weeks, or averages across weeks.

The intent of the bar graphs referred to by the reviewer in this point (Fig. 1E and 1F) was not subjectively chosen to show greater effects of one treatment over another. We have now added the following to the revised Results section, lines 410-414: “In addition to data plotted over the entire experimental time course, WK3 and WK6 data are also shown in individual bar graphs, as these time points in the behavioural testing regimen represent the animals’ responses to the von Frey test one week and 4 weeks (one month), respectively, after daily treatment ceased (Fig. 1E and 1F).”

c. When statistically significant, F and P values of the 2-way ANOVA’s are required.

F and P values have now been added to the revised Results, where appropriate.

d. The manuscript Results must be very clear to state that the data of Fig 1 and Fig 2 are the same data, just plotted differently. This should also be stated in the figure caption to Fig 2.

This point has now been addressed in the revised Results section, lines 444-445: “Data in Fig. 1 was replotted in Fig. 2 to examine sex differences across each cannabinoid treatment regimen.” The revised legend for Fig. 2 also acknowledges this point (lines 469-470).

e. Much of the information in the Figure Captions belongs in the Methods.

The figure captions have been edited, especially for Fig. 3 (lines 519-545), which included data that had already been presented in Tables 5 and 6 in the previously submitted Methods section.

f. For Fig 3, “CUT” vs not cut neurons are not defined. Explain why only delineated for A-beta? Were CUT neurons sensitized as compare to “MS” neurons – statistical comparison may be useful here. Discussion should compare this difference to the literature.

The definition of a “CUT” neuron corresponds to what we have routinely used in our previous publications. Please refer to these references and the revised Methods section, lines 279-300, which explains how these neurons were classified. Specifically, based on action potential (AP) configuration, conduction velocity (CV), and receptive field properties defined using hand-held mechanical stimulators, recorded neurons were classified as C-type high-threshold mechanosensitive fibres (CHTM), Aβ-type high-threshold mechanosensitive fibres (Aβ HTM), or Aβ-type low-threshold mechanosensitive fibres (Aβ LTM). Other major factors were used to further classify Aβ LTM neurons as either cutaneous (CUT) or muscle spindle (MS) neurons. MS neurons were slowly adapting neurons with deep subcutaneous receptive fields activated by deep tissue manipulation of the muscle belly but not by cutaneous stimulation. Thus, those neurons receiving high threshold cutaneous stimulation will belong to CHTM or AβHTM. However, a “CUT” neuron was only delineated by Aβ LTM.

Please note that the revised Discussion now includes a paragraph that addresses this point, as requested by the reviewer; see lines 716-731.

g. Fig 3A does not make sense. For example, 4000 pA amplitude is not shown. The authors must explicitly state which groups are different, as designated by upper or lower case letters. Difference from vehicle? Again, statistics results of 2-way ANOVA are required.

We thank the reviewer for pointing out that the 4000 pA amplitude was not shown; we have corrected Fig. 3A, which now correctly shows the application of current injections of 100 ms each, delivered with an amplitude of 500 to *4000 pA* in increments of 500 pA.

We have revised the data in Fig. 3C-F to visually present the graphs in a different format. In the updated version, the letters for each multiple comparison have been replaced with bars and stars between groups that were found to be significant via 1-way ANOVA (Kruskal-Wallis test with a Dunn’s multiple comparison post hoc analysis). We have included data for age-matched male and female naïve animals (no surgery) for each comparison, as we unfortunately do not have equivalent sham data for either sex at endpoint. Please note that in the revised Results section, each group that is statistically significant from another group has been thoroughly described.

h. The data of Figure 4 would be much more impactful if data SHAM vs INJURED are shown. Why not? If undetectable levels in SHAM then this should be reported. It is very important to show whether injury changes any of these T cell markers, and to discuss whether any such change is reversed or enhanced by the drugs. Again, F and p value results of ANOVA are required in the Results section.

We did have a reason why sham data was not included in Fig. 4 of the original submission: only two independent tissue sample sets (thymus, spleen, and DRGs) had been collected for the male sham group (i.e. from n=2 individual animals). qPCR data graphed in Fig. 4 now includes relative mRNA levels of male and female sham-operated animals, in addition to all the other groups (VEH, THC, CBD, and Combo). The n number for each tissue is clearly indicated in Table 1 (line 333).

We hope that the small sample size for the male sham group is acceptable to the reviewer, given that it is currently not possible to repeat this >9-week experiment due to our lab’s closure in response to the Covid-19 crisis. We have recalculated all the qPCR results using the delta delta Ct method, set relative to the male sham-treated group. Please note that F and P values for all ANOVAs, for each marker in each tissue, are now shown in the revised Results section.

***3. The Discussion requires organization.***

Use informative subheadings. One key conclusion per paragraph, please.

We have incorporated informative subheadings (*Cannabinoid-Mediated Effects on Mechanical Nociceptive Behaviour*, *The Effects of Cannabinoid Treatment on Fiber-Specific Neurotransmission*, *Changes in Immune Marker Profiles*, and *Summary*) into the revised Discussion. There are now one or two key conclusions that are addressed per subsection/paragraph. We hope that this organization is acceptable.

The discussion of T-cells is far too long and speculative.

We have shortened the revised Discussion with regard to T cells, with less speculation. Please note that this section has undergone significant changes – while it remains lengthy, it includes pertinent discussion of relevant literature in the context of the presented results.

a. Line 1 of the Discussion seems to state that THC and CBD are associated with pain chronification. Isn’t it the opposite?

We appreciate how the reviewer may have misinterpreted this sentence due to its original wording in the initial submission. We have changed it to read: “THC and CBD both play roles in modulating neurotransmission and immune function. When a nerve is injured or damaged, nociceptive pathways and the immune system are affected, and the dysregulation of these neuroimmune processes has been associated with pain chronification.” Please see lines 652-655 of the revised Discussion.

b. p.23 discussion of neural repair is much too lengthy in the absence of any data on neural repair.

We have significantly shortened the original Discussion with regard to neural repair.

c. P.24 indicates that the literature shows higher levels of T cells in females. This contradicts most of the data shown in Figure 4. The authors speculate this is due to the nerve injury – the authors ran Sham injured rats and should compare SHAM vs CUT data to address their speculation.

The literature discussed on page 24 of the originally submitted manuscript does not contradict the initially submitted data shown in the original Fig. 4, as it refers to sex differences in T cell levels prior to any injury/damage (a pre-primed basal state). However, we have now added sham data for male and female rats, which will clarify this point.

Although we carried out behavioural von Frey testing and collected tissue samples for the male and female sham groups, we unfortunately did not conduct electrophysiology on these animals at the 9-week experimental endpoint. We apologize that we do not have this data set, and hope that this will not be a deciding factor against publishing our revised study. We did, however, have unpublished data for naïve male and female rats that were electrophysiologically profiled for the same set of neurons at the level of DRG soma in age-matched rats, which we have now included in Fig. 3C-F and used to discuss our nerve injury data.

***4. The Discussion must relate the findings of Figs 1-3 to the existing literature.***

This must be done thoroughly, with greatest attention to the strongest findings.

We hope that we have done this satisfactorily in the revised Discussion. Please refer to the updated Discussion, which has related our primary findings to the existing literature.
